# Reversal of Ophthalmic Artery Flow and Stroke Outcomes in Asian Patients with Acute Ischemic Stroke and Unilateral Severe Cervical Carotid Stenosis

**DOI:** 10.1371/journal.pone.0080675

**Published:** 2013-12-02

**Authors:** Yueh-Feng Sung, Chia-Lin Tsai, Jiunn-Tay Lee, Chi-Ming Chu, Chang-Hung Hsu, Chun-Chieh Lin, Giia-Sheun Peng

**Affiliations:** 1 Graduate Institute of Medical Sciences and Department of Neurology, Tri-Service General Hospital, National Defense Medical Center, Taipei, Taiwan, Republic of China (R.O.C.); 2 Department of Neurology, Tri-Service General Hospital, National Defense Medical Center, Neihu District, Taipei, Taiwan, R.O.C.; 3 Department of Epidemiology, School of Public Health, National Defense Medical Center, Taipei, Taiwan, R.O.C.; Banner Alzheimer's Institute, United States of America

## Abstract

**Background:**

The aim of this study was to assess the clinical implications of reversed ophthalmic artery flow (ROAF) for stroke risk and outcomes in subjects with unilateral severe cervical carotid stenosis/occlusion.

**Methods:**

We investigated 128 subjects (101 with acute stroke and 27 without), selected from a large hospital patients base (n  =  14,701), identified with unilateral high-grade cervical carotid stenosis/occlusion by using duplex ultrasonography and brain magnetic resonance imaging. All clinical characteristics were compared for stroke risk between acute stroke and nonstroke groups. Patients with acute stroke were divided into 4 subgroups according to ophthalmic artery flow direction and intracranial stenosis severity, and stroke outcomes were evaluated.

**Results:**

The acute stroke group had significantly higher percentages of ROAF (52.5%, p  =  0.003), carotid occlusion (33.7%, p  =  0.046), and severe intracranial stenosis (74.3%, p<0.001). However, multivariate analysis demonstrated that intracranial stenosis was the only significant risk factor (odds ratio  =  10.38; 95% confidence interval  =  3.64–29.65; p<0.001). Analysis of functional outcomes among the 4 subgroups of patients with stroke showed significant trends (p  =  0.018 to 0.001) for better stroke outcomes from ROAF and mild or no intracranial stenosis. ROAF improved 10–20% stroke outcomes, as compared to forward ophthalmic artery flow, among the patients with stroke and the same degree of severities of intracranial stenosis.

**Conclusions:**

Patients with acute stroke and severe unilateral cervical carotid stenosis/occlusion significantly have high incidence of intracranial stenosis and ROAF. Intracranial stenosis is a major stroke risk indicator as well as a predictor for worse stroke outcomes, and ROAF may provide partial compensation for improving stroke outcomes.

## Introduction

Extracranial severe carotid stenosis or occlusion is a well-known pathogenic factor for ischemic stroke. The risk of stroke increases in patients with concurrent severe stenosis of extra- and intracranial vessels [1], [2]. Their cerebral collaterals are recruited to compensate for diminished intracranial blood flow to maintain cerebral homeostasis. The cerebral collaterals include the circle of Willis as the primary collateral pathway and extracranial reversed ophthalmic artery flow (ROAF) as the secondary collateral. ROAF can occur as a result of intracranial hemodynamic compromise with insufficient collaterals from the circle of Willis. The phenomenon of ROAF is well known [3], but its clinical implication in patients with severe cervical carotid stenosis/occlusion remains poorly understood. Previous studies [3]–[9] have demonstrated that ROAF, a sign of deteriorated hemodynamic status in the brain, is often associated with impaired cerebral vasoreactivity and is liable to subsequent cerebral ischemic events. We previously demonstrated that patients with concurrent stenosis of extra- and intracranial vessels are more likely to have ROAF and a poor functional outcome [10]. However, the effect of ROAF per se on stroke outcomes in patients with acute ischemic stroke and severe unilateral cervical carotid stenosis/occlusion remains unclear.

To address this question, we selected patients with acute ischemic stroke and unilateral high-grade cervical carotid stenosis/occlusion and evaluated clinical functional outcomes in the presence of ROAF and intracranial stenosis. We hypothesized that intracranial stenosis would be a predictor for stroke risk and poor stroke outcomes and that the presence of ROAF would provide compensation for improving stroke outcomes in patients with ischemic stroke and severe unilateral cervical carotid stenosis/occlusion.

## Patients and Methods

### Selection of study patients and clinical characteristics

**Figure 1:**
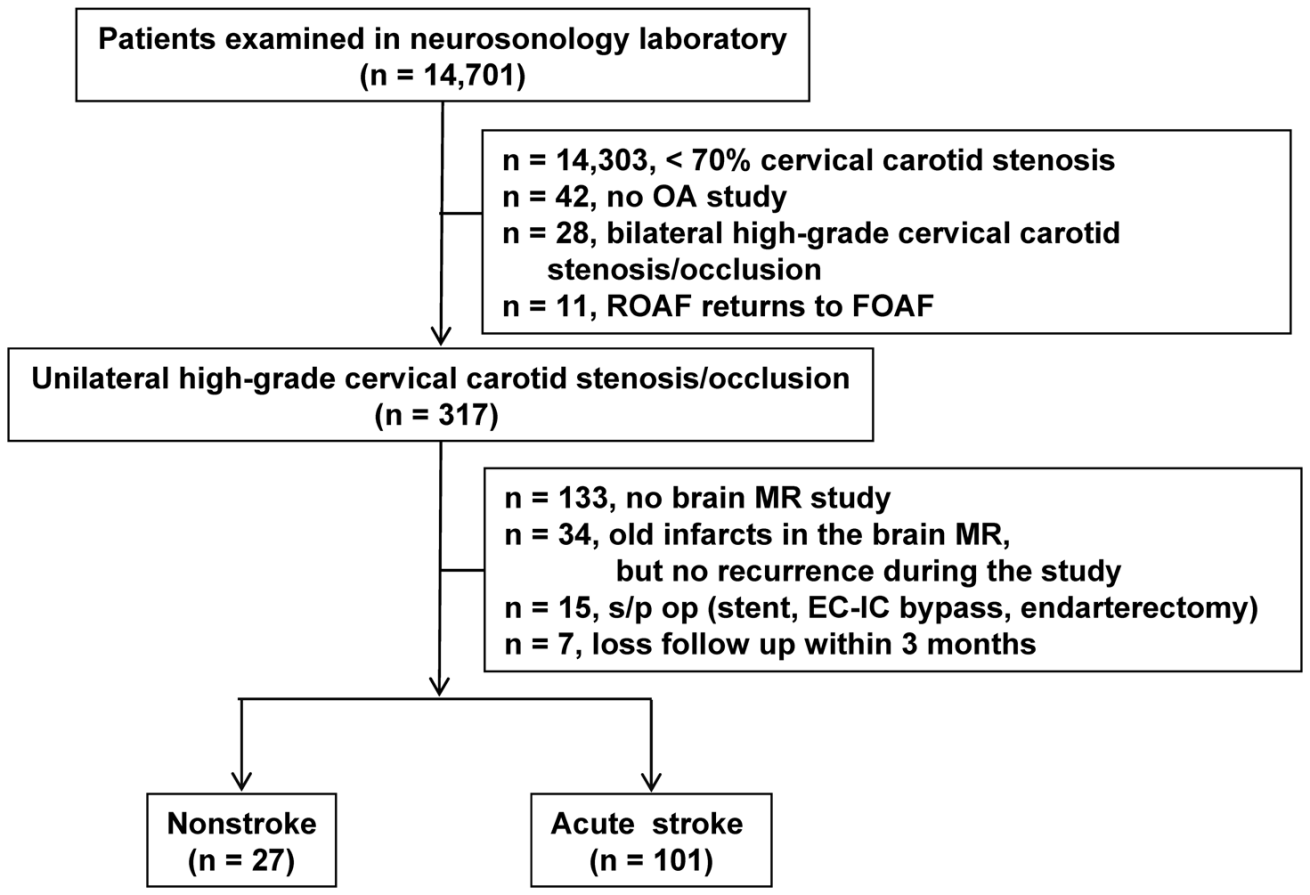
Study patient selection. FOAF, forward ophthalmic artery flow; MR, magnetic resonance; ROAF, reversed ophthalmic artery flow; EC-IC bypass, extracranial–intracranial bypass.

This retrospective cohort study was carried out within the certificated Neurovascular Ultrasound Laboratory in Tri-Service General Hospital, Taipei, Taiwan. Written consent was given by every subject before the examination. The study was approved by the hospital's Institutional Review Board for Human Studies. Between January 1, 2005 and July 31, 2012, 3 certified sonographers screened 14,701 consecutive subjects for carotid stenosis (for selection scheme of study patients, see Figure 1). Each individual underwent color-coded duplex ultrasonography (CCDU) of the cervical and retrobulbar vessels with an ATL HDI 5000 ultrasound system (Philips, Bothell, WA, USA) with a L12–5 linear 38-mm transducer. An internal carotid artery (ICA) with 70–99% diameter reduction or total occlusion was diagnosed according to the laboratory's ultrasound criteria [11], which have been validated to correspond to the degree of stenosis on cerebral angiography measured by the European Carotid Surgery Trial method [12] and with carotid endarterectomy findings. The degree of luminal narrowing was calculated from the ratio of the minimal residual diameter of the stenosed segment to the presumed former diameter of the same segment [12]. Blood flow direction in the ophthalmic artery (OA) was categorized as forward if it was directed away from the stenotic ICA or as reversed if it was directed toward the stenotic ICA. Peak velocities, end-diastolic velocities, and resistive indexes of the ophthalmic and central retinal arteries, cervical common carotid artery, ICA, and external carotid artery were routinely measured. The entire process for each subject was recorded with a digital video system, and examination data were checked by 2 neurovascular specialists.

After excluding 14,303 patients without high-grade carotid stenosis, 42 without OA examination, 28 with bilateral high-grade carotid stenosis/occlusion, and 11 with ROAF remission, 317 symptomatic or asymptomatic subjects were diagnosed with unilateral high-grade stenosis (70–99%) or occlusion (100%) of the cervical segment of the ICA. They underwent a detailed neurological examination and provided clinical history based on a structured checklist [13] that was related to major vascular risk factors, such as hypertension, diabetes, hypercholesterolemia, hypertriglyceridemia, coronary artery disease, atrial fibrillation, previous stroke, and smoking. Among them, 133 without brain magnetic resonance imaging (MRI), 34 with old stroke but no recurrence, 15 who had undergone surgery for carotid stenosis, and 7 who were lost during follow-up were excluded. Ultimately, 128 Asian patients with unilateral high-grade cervical carotid stenosis/occlusion were recruited from the neurosonography laboratory dataset into this study (Figure 1). Among them, only 27 patients had severe unilateral cervical carotid stenosis/occlusion without stroke and were included as the non-stroke control group, and 101 had acute symptomatic stroke, which was diagnosed according to typical stroke symptoms and positive brain MRI findings. Twenty-seven subjects in the non-stroke group were referred for neurosonological evaluation owing to 14 with dizziness; 7 carotid bruits; 4 transient ischemic attack and 2 syncope. The acute stroke study group comprised 58 patients with first-ever acute ischemic strokes and 43 with recurrent acute cerebral infarctions.

### Grade of intracranial stenosis and outcome measures

Head MRI (Signa HDx; GE Healthcare, Waukesha, WI, USA) was performed to differentiate stroke type when clinically indicated. The 128 selected patients underwent cranial MRI with multiple modalities, including fluid-attenuated inversion recovery, diffusion-weighted imaging, and three-dimensional time-of-flight magnetic resonance angiography, to differentiate stroke pattern and type if appropriate. The grade of intracranial vascular stenosis was classified into 2 groups: normal to mild stenosis (≤50%) or moderate stenosis to occlusion (>50%), as described in previous reports [11], [13], [14]. Functional outcomes were measured using the modified Rankin Scale (mRS) and the National Institutes of Health Stroke Scale (NIHSS) at stroke onset and 3 months later, respectively. A mRS score of 0 to 3 and an NIHSS score of 0 to 6 was defined as a good clinical outcome for comparing the impact of intracranial stenosis and OA flow direction. To evaluate the implicit role of intracranial stenosis and OA flow direction in stroke outcomes, patients with acute stroke were divided into 4 subgroups: severe intracranial stenosis (>50%) and forward OA, severe intracranial stenosis (>50%) and reversed OA, mild intracranial stenosis (≤50%) and forward OA, and mild intracranial stenosis (≤50%) and reversed OA.

### Statistical analysis

Continuous variables were calculated as mean ± standard deviation. Categorical variables were expressed as numbers and percentages. Categorical or continuous variables were patient age, gender, vascular risk factors, ROAF, and cervical and intracranial stenosis. Odds ratios (ORs) were used to estimate multivariate relative risks, and 95% confidence intervals (CIs) were evaluated with age-adjusted logistic regression analyses. The multivariate logistic regression with a forward selection model was performed to identify significantly independent contribution factors for acute stroke. Attributable risk difference was calculated between the acute stroke and non-stroke groups for investigating the risk of ROAF in patients with acute stroke and severe intracranial stenosis. Fisher's exact tests for categorical variables and Mantel–Haenszel extension tests for trend analyses were conducted for stroke outcome evaluation to assess the compensation effect of ROAF on intracranial stenosis severity. Statistical analyses were performed with SPSS version 19 (SPSS, Chicago, IL, USA). Values were considered statistically significant at p<0.05.

## Results

Out of 14,701 consecutive subjects, only 128 met the study criteria. Among them, 27 subjects had not experienced strokes and had no evidence of stroke as assessed clinically and with brain MRI, and these subjects were considered as the non-stroke group. The remaining 101 subjects had experienced acute ischemic strokes, including 58 with first-ever onset and 43 with recurrent strokes. There were no statistically significant differences in clinical characteristics after age-adjusted univariate analysis between the 2 subgroups, and they were hence collectively considered as the acute stroke group. Statistical analysis revealed that acute stroke group was younger (68.7±13.7 vs. 76.6±7.6, p<0.001) than the non-stroke group. The reason may be that the lack of symptoms in the non-stroke group delayed their decision to screen for the risk of carotid stenosis. To further determine acute stroke risk factors in patients with unilateral high-grade cervical carotid stenosis/occlusion, we compared clinical characteristics between the non-stroke and acute stroke groups with age-adjusted univariate logistic analysis. Table 1 shows that the acute stroke group had a significantly higher percentage of ROAF (52.5%, p  =  0.003), carotid occlusion (33.7%, p  =  0.046), and intracranial stenosis (74.3%, p<0.001).

**Table 1:** Age-adjusted analysis of clinical characteristics in acute strokes with unilateral high-grade cervical internal carotid stenosis or occlusion.

	Nonstroke	Acute stroke	P-value	OR	95% CI
Number of subjects (%)	27 (21.1)	101 (78.9)			
Age, mean ± SD, year	76.6±7.6	68.7±13.7			
Sex (M:F)	19∶8	73∶28	0.862	0.92	0.35–2.43
Risk factors, no. (%)					
Hypertension	23 (85.2)	79 (78.2)	0.953	1.04	0.30–3.57
DM	6 (22.2)	42 (41.6)	0.079	2.48	0.90–6.83
Hypercholesterolemia	9 (33.3)	34 (33.7)	0.691	0.83	0.32–2.13
Hypertriglyceridemia	2 (7.4)	12 (11.9)	0.696	1.38	0.28–6.86
CAD	11 (40.7)	18 (17.8)	0.072	0.42	0.16–1.08
AF	1 (3.7)	17 (16.8)	0.073	6.86	0.83–56.47
Smoking	8 (29.6)	53 (52.5)	0.087	2.28	0.89–5.84
Reversed OA flow	4 (14.8)	53 (52.5)	0.003	5.65	1.79–17.86
Carotid stenosis, no. (%)			0.046	3.80	1.02–14.08
70–99% stenosis	24 (88.9)	67 (66.3)			
Occlusion	3 (11.1)	34 (33.7)			
Intracranial stenosis, no. (%)					
>50%	6 (22.2)	75 (74.3)	<0.001	10.38	3.64–29.65

AF, atrial fibrillation; CAD, coronary artery disease; CI, confidence interval; DM, diabetes mellitus; F, female; M, male; NIHSS, National Institutes of Health Stroke Scale; no, number; OA, ophthalmic artery; OR, odds ratio. Statistically significant differences for categorical variables between tested groups were evaluated using age-adjusted logistic analysis.

Among the 81 patients with intracranial stenosis (6 in non-stroke, 75 in acute stroke), 80 had ipsilateral severe intracranial stenosis/occlusion in the anterior circulation, and 33 patients had other concomitant critical lesions in the contralateral anterior or posterior circulation. However, no difference of ROAF occurrence was observed between patients with ipsilateral severe intracranial stenosis/occlusion in the anterior circulation and those with multiple severe intracranial stenoses at other locations.

To evaluate the roles of individual risk factors in predisposition to acute stroke in patients with unilateral high-grade cervical carotid stenosis/occlusion, we performed multivariate analyses with a forward selection model (Table 2). The comparison with the non-stroke group, including all the confounding factors except for the variable of intracranial stenosis as model 1, showed that patients with acute ischemic stroke had higher prevalence rates of ROAF (OR  =  6.50; 95% CI  =  2.00–21.11; p  =  0.002) and DM (OR  =  3.08; 95% CI  =  1.06–8.90; p  =  0.038) than did those in the non-stroke group. When all the confounding factors were included in the comparison as model 2, the acute ischemic stroke group had a significantly higher occurrence of intracranial stenosis (OR  =  10.38; 95% CI  =  3.64–29.65; p<0.001) than did the non-stroke group, but the effects of ROAF and DM became insignificant in this model.

**Table 2:** Multivariate analysis of risk factors for acute stroke in patients with unilateral high-grade cervical internal carotid stenosis or occlusion.

	Patient no.(*n* = 128)	Acute stroke	P-value	AdjustedOR	95% CI	R^2^
Model 1						0.28
ROAF						
Yes	57	53	0.002	6.50	2.00–21.11	
No	71	48		1.00		
DM						
Yes	48	42	0.038	3.08	1.06–8.90	
No	80	59		1.00		
Model 2						0.35
Intracranial stenosis						
>50%	81	75	<0.001	10.38	3.64– 29.65	
≤50%	47	26		1.00		

CI, confidence interval; DM, diabetes mellitus; no., number; OR, odds ratio; ROAF, reversed ophthalmic artery flow.

Model 1: Statistically significant difference determined using multivariate logistic regression with forward selection model controlled for age, gender, vascular risk factors, and cervical stenosis but not including the variable of intracranial stenosis.

Model 2: Statistically significant difference determined using multivariate logistic regression with forward selection model controlled for age, gender, vascular risk factors, cervical stenosis, and ophthalmic artery flow direction.

We employed logistical regression analysis to explore the role of severe intracranial stenosis in ROAF occurrence. Table 3 shows that the subjects with severe intracranial stenosis were at increased risk for ROAF (OR  =  3.38 in the acute stroke group, OR  =  4.75 in the non-stroke group). The attributable risk was 1.37, but there was no significant difference between the two groups.

**Table 3:** Attributable risk difference of ROAF in acute strokes with unilateral high-grade cervical internal carotid stenosis/occlusion and intracranial stenosis.

	Acute stroke, no.		OR	95% CI	Nonstroke, no.		OR	95% CI	Attributable risk
	OA flow				OA flow				
	Reversed	Forward			Reversed	Forward			
Intracranial stenosis, no.									
>50%	45	30	3.38	1.30–8.75	2	4	4.75	0.51–44.48	1.37
≤50%	8	18			2	19			

CI, confidence interval; no., number; OR, odds ratio; ROAF, reversed ophthalmic artery flow.

**Figure 2:**
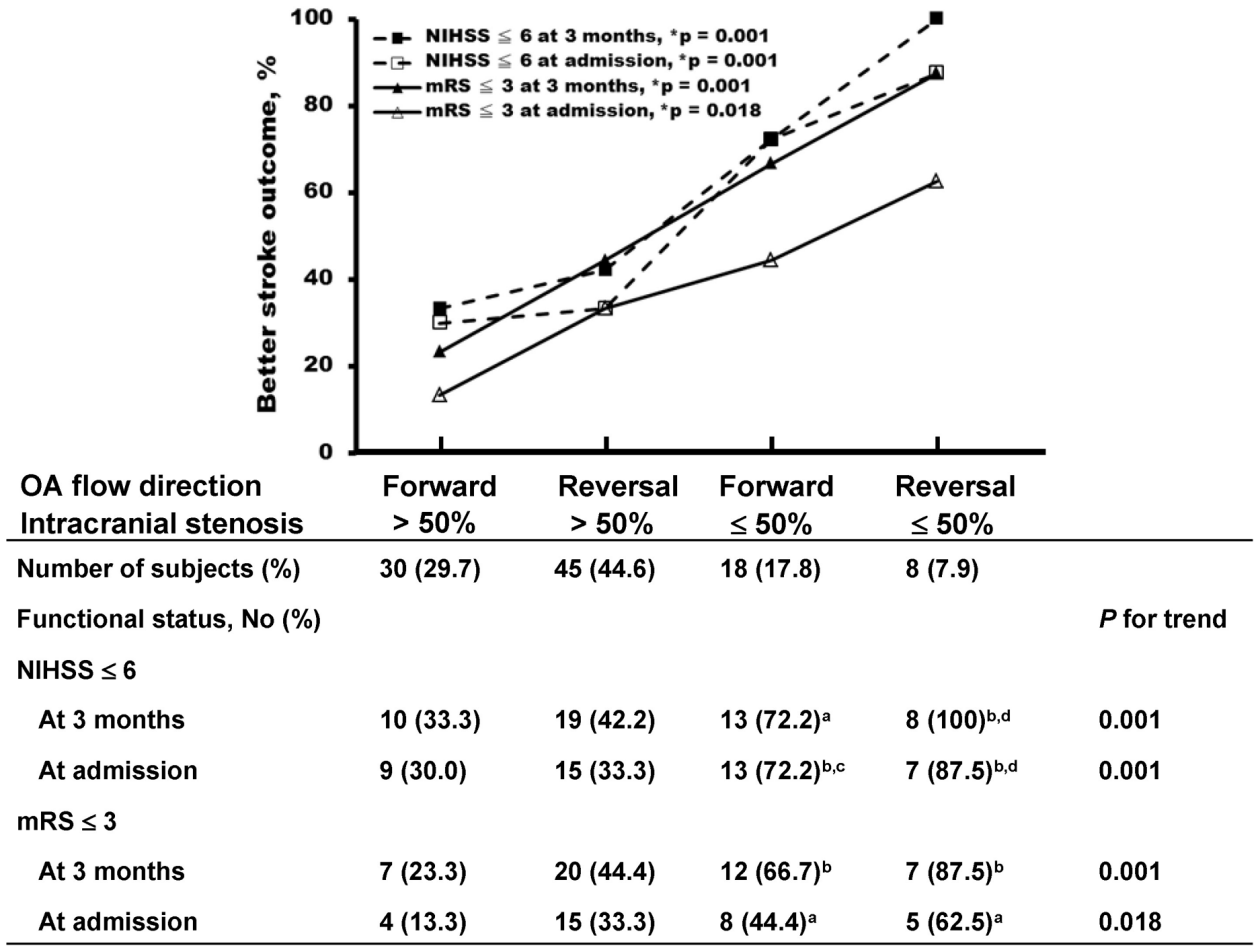
Functional outcomes of patients with acute stroke in the presence of ROAF or intracranial stenosis. Patients with acute stroke were divided into 4 subgroups: severe intracranial stenosis (>50%) and forward OA, severe intracranial stenosis (>50%) and reversed OA, mild intracranial stenosis (≤50%) and forward OA, and mild intracranial stenosis (≤50%) and reversed OA.ROAF and less intracranial stenosis are good predictors for acute stroke outcomes. mRS, modified Rankin Scale; NIHSS, National Institutes of Health Stroke Scale; no., number; OA, ophthalmic artery; *p = p value for trend. Statistically significant differences were evaluated using Fisher's exact test for categorical variables between the tested groups and Mantel–Haenszel extension tests for trend analyses. ^a^p<0.05 vs. forward OA and intracranial stenosis >50%; ^b^p<0.01 vs. forward OA and intracranial stenosis >50%; ^c^p<0.05 vs. reversed OA and intracranial stenosis >50%; ^d^p<0.01 vs. reversed OA and intracranial stenosis >50%.

To evaluate the relationship between OA flow direction and intracranial stenosis severity in the context of stroke outcomes, 4 acute stroke subgroups were analyzed by Fisher's exact tests for categorical variables and Mantel–Haenszel extension tests for trend analyses. However, regardless of blood flow direction of OA, the patients with intracranial stenosis had worse stroke outcomes than those without. Figure 2 illustrates the functional status among the 4 groups at the time of stroke onset and 3 months later. The better stroke outcomes among the 4 groups corresponded to the following order: severe intracranial stenosis/occlusion and forward ophthalmic artery flow (FOAF), severe intracranial stenosis/occlusion and ROAF, mild intracranial stenosis and FOAF, and mild intracranial stenosis and ROAF by a significant increase of better stroke outcome scores at ratios of 10–20%, that is, p  =  0.001, 0.001, 0.001, and 0.018 for NIHSS ≤6 at 3 months, NIHSS ≤6 at admission, mRS ≤3 at 3 months, and mRS ≤3 at admission, respectively, for trend of the outcomes (Figure 2). Interestingly, the reversal of OA flow always improved stroke outcomes by 10–20%, as compared to FOAF in patients with the same severity of intracranial stenosis.

## Discussion

This study demonstrated that intracranial stenosis was the major risk factor for the development of acute stroke in patients with severe unilateral cervical carotid stenosis/occlusion. Patients with acute ischemic stroke and concurrent severe cervical carotid stenosis/occlusion and intracranial stenosis had a higher rate of ROAF and worse stroke outcomes compared to those with extracranial severe carotid stenosis alone. However, ROAF partially compensated for cerebral insufficiency and was associated with improved stroke outcome.

Angiographic and pathologic evidence has previously demonstrated racial differences in the distribution of cervicocerebral atherosclerosis [15]–[18]. East Asians have higher rates of intracranial stenosis, whereas Caucasians have higher rates of extracranial stenosis [17], [19]–[21]. Meanwhile, an increasing body of evidence suggests that intracranial atherosclerotic disease is both a major cause of ischemic stroke and an independent risk factor for subsequent stroke in patients on conservative medical treatment with symptomatic ICA stenosis [1], [10], [19], [22]–[24]. Previous studies have shown that the occurrence of intracranial stenosis was detected in 33–37% of Chinese patients with ischemic stroke [23], [25] and in 69–84% of Indian patients with large-artery stroke [2], [26], [27]. Concurrent atherosclerosis of intra- and extracranial cervicocerebral vessels is relatively common in Asian populations [1], [17], [18], [25], [28], [29]; approximately 20–50% of patients with extracranial stenosis also showed evidence of intracranial stenosis [10], [22], [29], [30]. This study (Table 1) showed a 74.3% incidence of intracranial stenosis in the stroke group and 22.2% in the non-stroke group, a finding in agreement with those of previous studies.{Tsai, 2013 #197}{Tsai, 2013 #197} {Tsai, 2013 #197}Multivariate logistic regression with forward selection model (Table 2) revealed that the patients with concurrent severe extra- and intracranial stenosis have tenfold stroke risk as compared to those without intracranial stenosis. If intracranial stenosis was not taken into account, ROAF had a sixfold increased stroke risk in patients with unilateral severe cervical carotid stenosis/occlusion. Thus, intracranial stenosis was a more important predictor of stroke risk than ROAF in patients with unilateral severe cervical carotid stenosis. Previous reports indicated that patients with symptomatic stroke and severe cervical carotid stenosis should be allocated to surgery [12] or stenting [31] irrespective of the presence of ROAF or FOAF. However, our preliminary data [10] and others [32] showed that ROAF can be corrected to forward flow after endarterectomy or stenting, suggesting the dynamic role of ROAF depending on the severity of intracranial compromise. The appearance of ROAF in patients with stroke and unilateral severe cervical carotid stenosis who do not receive surgery suggests it may be an indicator of impaired intracranial hemodynamics, indicating that the patients were at higher risk for stroke and would need more aggressive intervention.

In Table 3, the patients with concurrent severe cervical and intracranial stenosis had a significantly higher prevalence of ROAF, as compared to those with severe cervical carotid stenosis/occlusion alone irrespective of being in the stroke group or the nonstroke group. Interestingly, the occurrence of ROAF in the patients without stroke and with concurrent severe cervical and intracranial stenosis was higher than that in the patients with stroke and concurrent severe cervical and intracranial stenosis; however, the differences were not significant. The result suggested that ROAF might modify stroke risk in patients with concurrent severe atherosclerosis of the intra- and extracranial cerebrovascular vessels. Recent studies [33]–[35] demonstrated that the extent and number of collateral circulation pathways are a potent determinant of stroke risk and outcome in patients with intracranial stenosis. Most data suggested that the compensatory effect was from the primary cerebral collaterals, but no sufficient data had been obtained from the secondary collateral, ROAF [33]–[35]. Our previous study [10] demonstrated that concurrent severe atherosclerosis of the intra- and extracranial cerebrovascular vessels appears to be a significant risk factor for poor functional outcome and increased incidence of ROAF, and the presence of ROAF may result from intracranial hemodynamic compromise. To date, there was insufficient data to determine the role of ROAF per se in stroke outcomes of patients with acute ischemic stroke and concurrent extra- or intracranial atherosclerosis. Figure 2 showed that intracranial stenosis was a major determinant of worse stroke outcome in patients with acute ischemic stroke. Patients with concurrent severe atherosclerosis of the intra- and extracranial cerebrovascular vessels had worse stroke outcomes than those without intracranial stenosis. In the subjects with the same severity of intracranial stenosis, stroke outcome improved 10–20% in the ROAF group compared to the FOAF group, suggesting that ROAF exerts partial hemodynamic compensation for improvement of the stroke outcomes in patients with acute ischemic stroke and unilateral high-grade cervical stenosis/occlusion. This partial compensation effect of ROAF may provide a reason why patients with stroke and ROAF have no complete protection against stroke recurrence. The partial compensation may be ascribed to the smaller caliber and lower blood flow in the OA compared to the ICA. Previous studies have demonstrated that the ICA diameter was 0.44–0.55 cm compared to 0.11–0.20 cm for the OA. Blood flow was 215.6–317.6 mL/min in the ICA and 6.8–9.7 mL/min in the OA [36], [37]. The diameter and blood flow of the vertebral artery was 0.26–0.35 cm and 44.8–85.5 mL/min, respectively [36]. It is obvious that compensation from OA is much less efficient than the primary collaterals, which are derived from contralateral ICA or vertebral arteries. Therefore, patients with stroke and unilateral high-grade cervical carotid stenosis/occlusion may have some compensation from ROAF, but they still exhibit relative cerebral hypoperfusion and thus appear to be at long-term risk for recurrent stroke. A previous study [8] demonstrated that patients with ROAF had more subsequent cerebral ischemic events than those with forward flow, suggesting that ROAF was indicative of poor stroke outcome. However, their study did not assess potential interactions between ROAF and intracranial stenosis identified in this study. Indeed, the present study has revealed a partial benefit from ROAF for stroke outcome scores by the trend analysis of 4 subgroups of patients with varying intracranial stenosis and ROAF/FOAF (Fig. 2). In future studies, it will require much larger sample size for each patient subgroup to increase statistical power to further clarify causal interrelations between severities of intracranial stenosis and the ophthalmic artery flow directions in relation to benefit of stroke outcomes.

The strengths of this study are that the included patients were strictly selected using CCDU and brain MR angiography data from a large patient pool (n  =  14,701), and the patients with ischemic stroke all had the large artery atherosclerosis stroke type. However, the novel findings and potential implications of ROAF are limited by the lack of conventional digital subtraction angiography, which is the gold standard for confirming the diagnosis of extra- and intracranial stenosis and for imaging the cerebral collateral vessels. In addition, some medications in patients with ischemic stroke have been linked to better collateral circulation [38]; therefore, previous history of medications before stroke is another influential determinant for the presence of collaterals that was not included in the present study.

## Conclusion

Our study found that patients with acute ischemic stroke and unilateral severe cervical carotid stenosis/occlusion had a significantly higher occurrence of severe intracranial stenosis and ROAF. Regardless of blood flow direction of OA, the patients with severe intracranial stenosis have worse stroke outcomes as compared to those without. ROAF may act as a secondary collateral when brain tissues receive insufficient blood supply from the primary collaterals, thereby providing patients a valuable, but limited, compensation for partial improvement of stroke outcomes. The results of this study revealed an important implication for clinical practice. It suggested that patients with ischemic stroke should undergo advanced brain imagining and cerebral hemodynamic evaluation when they were detected with unilateral severe cervical carotid stenosis/occlusion and ROAF by CCDU. Early identification of the intracranial pathological vascular lesions and hemodynamic compromise are essential for determining optimal therapeutic strategies for improving stroke outcomes.
